# Unravelling Faecal Microbiota Variations in Equine Atypical Myopathy: Correlation with Blood Markers and Contribution of Microbiome

**DOI:** 10.3390/ani15030354

**Published:** 2025-01-26

**Authors:** Anne-Christine François, Carla Cesarini, Bernard Taminiau, Benoît Renaud, Caroline-Julia Kruse, François Boemer, Gunther van Loon, Katrien Palmers, Georges Daube, Clovis P. Wouters, Laureline Lecoq, Pascal Gustin, Dominique-Marie Votion

**Affiliations:** 1 Department of Functional Sciences, Faculty of Veterinary Medicine, Pharmacology and Toxicology, Fundamental and Applied Research for Animals & Health (FARAH), University of Liège, 4000 Liège, Belgium; benoit.renaud@uliege.be (B.R.); p.gustin@uliege.be (P.G.); dominique.votion@uliege.be (D.-M.V.); 2 Equine Clinical Department, Faculty of Veterinary Medicine, Fundamental and Applied Research for Animals & Health (FARAH), University of Liège, 4000 Liège, Belgium; ccesarini@uliege.be (C.C.); laureline.lecoq@uliege.be (L.L.); 3 Department of Food Sciences–Microbiology, Faculty of Veterinary Medicine, Fundamental and Applied Research for Animals & Health (FARAH), University of Liège, 4000 Liège, Belgium; bernard.taminiau@uliege.be (B.T.); georges.daube@uliege.be (G.D.); 4 Department of Functional Sciences, Faculty of Veterinary Medicine, Physiology and Sport Medicine, Fundamental and Applied Research for Animals & Health (FARAH), University of Liège, 4000 Liège, Belgium; caroline.kruse@uliege.be; 5 Biochemical Genetics Laboratory, CHU, University of Liège, 4000 Liège, Belgium; f.boemer@chuliege.be; 6 Department of Internal Medicine, Reproduction and Population Medicine, Faculty of Veterinary Medicine, Ghent University, 9820 Merelbeke, Belgium; gunther.vanloon@ugent.be; 7 De Morette Equine Clinic, 1730 Asse, Belgium; katrien.palmers@demorette.be

**Keywords:** equine atypical myopathy, microbiota, gut microbiota, faecal microbiota, microbiome, faecal microbiome, horses, equine, hypoglycin A, methylenecyclopropylacetyl-carnitine, MCPA-CoA, acylcarnitines, 16S rRNA gene sequencing, next generation sequencing, NGS, blood metabolites, toxin, poisoning

## Abstract

Hypoglycin A and methylenecyclopropylglycine are protoxins responsible for atypical myopathy in equids. These protoxins are converted into toxins that inhibit fatty acid *β*-oxidation, leading to blood accumulation of acylcarnitines and toxin conjugates, such as methylenecyclopropylacetyl-carnitine. The enzymes involved in this activation are also present in some prokaryotic cells, raising questions about the potential role of intestinal microbiota in the development of intoxication. Differences have been noted between the faecal microbiota of cograzers and atypical myopathy-affected horses. However, recent blood acylcarnitines profiling revealed subclinical cases among cograzers, challenging their status as a control group. This study investigates the faecal microbiota of horses clinically affected by atypical myopathy, their cograzers, and a control group of toxin-free horses while analysing correlations between microbiota composition and blood parameters. Faecal samples were analysed using 16S amplicon sequencing, revealing significant differences in *α*-diversity, evenness, and *β*-diversity. Notable differences were found between several genera, especially *Clostridia_ge*, *Bacteria_ge*, *Firmicutes_ge*, *Fibrobacter*, and *NK4A214_group*. Blood levels of methylenecyclopropylacetyl-carnitine and C14:1 correlated with variations in faecal microbial composition. The theoretical presence of enzymes in bacterial populations was also investigated. These results underscore the critical need to investigate the potential role of intestinal microbiota in this poisoning and may provide insights for developing prevention and treatment strategies.

## Simple Summary

Equine atypical myopathy is a severe intoxication caused by protoxins synthesised by certain maple trees, notably sycamore maple (*Acer pseudoplatanus*). These protoxins are activated into harmful toxins that disrupt lipid metabolism by inhibiting specific steps of fatty acid *β*-oxidation, leading to the accumulation of acylcarnitines in the blood. This activation process is catalysed mainly by specific mitochondrial enzymes, which are also present in some bacteria. Horses grazing in close proximity to affected animals have shown differences in their faecal microbiota composition, suggesting that the role of gut microbiota in atypical myopathy could be more substantial than previously understood. Recently, blood analyses have demonstrated the existence of subclinical cases among these cograzers. The present study compares the faecal bacteria of horses affected by atypical myopathy, their cograzers, and a group of toxin-free horses serving as a control group. Results show significant differences in faecal bacterial diversity and composition between groups, particularly for certain bacterial genera. Additionally, blood levels of specific compounds appear to be associated with these bacterial changes. The theoretical presence of the enzymes involved in the protoxin activation process was also studied. These results highlight the importance of comprehensively studying intestinal microbiota to better understand its role in this generally fatal poisoning.

## 1. Introduction

Equine atypical myopathy (AM) is a seasonal and highly fatal [1] intoxication caused by the ingestion and metabolism of two protoxins contained in some *Acer* spp., including *Acer pseudoplatanus* and *Acer negundo* [2,3]: methylenecyclopropylalanine, also known as hypoglycin A (HGA), [2] and methylenecyclopropylglycine (MCPrG) [4].

Both HGA and MCPrG are non-proteinogenic amino acids [5] that are not toxic *per se*. Their metabolism into toxic metabolites requires the action of enzymes, mainly located in the mitochondria, involved in branched-chain amino acids (BCAAs) catabolic pathway [6]. The first step is a transamination, catalysed by the branched-chain amino acid aminotransferase (BCAT) [6,7]. The second and irreversible step, catalysed by the branched-chain α-keto acid dehydrogenase complex (BCKDHc), is an oxidative decarboxylation with coenzyme A (CoA) added to oxidised products [6,8]. This results in the formation of methylenecyclopropylacetyl-CoA (MCPA-CoA) for HGA and methylenecyclopropylformyl-CoA (MCPF-CoA) for MCPrG [6]. These toxic metabolites impair the *β*-oxidation of fatty acids, leading to increased levels of acylcarnitine in tissues [9], urine [2,10,11] and blood [5,12,13,14,15,16]. The diagnosis of AM relies on a combination of clinical signs, acylcarnitine profiling, and the detection of toxic metabolites conjugated with carnitine or glycine, such as MCPA-carnitine [3,16,17,18,19].

Both prokaryotic and eukaryotic cells can metabolise amino acids [20]. Indeed, some bacteria have the above-mentioned enzymes: for example, *Lactococcus lactis* and *Escherichia coli* express BCAT [21,22], and *Bacillus subtilis*, *Staphylococcus aureus* and *Pseudomonas putida* express BCKDHc [23,24,25,26,27,28,29,30,31]. This raises the hypothesis that the intestinal microbiota may play a role in the metabolism of HGA and/or MCPrG, potentially affecting horses that have ingested these protoxins. Analysis of the faecal microbiota is of twofold interest in exploring this hypothesis: on top of being a non-invasive technique, the faecal microbiota is reflective of the colonic microbial population in equids [32,33,34].

Wimmer-Scherr et al. (2021) conducted a study exploring the differences in faecal microbiota between horses with AM and a control group consisting of their cograzers (CoG) (i.e., horses grazing in the same pasture as a poisoned horse but having a normal physical examination). They described that (1) the relative abundance of families *Ruminococcaceae*, *Christensenellaceae* and *Akkermansiaceae* was higher in horses suffering from AM compared to their CoG, and (2) the relative abundance of families *Lachnospiraceae*, *Bacteroidales* and *Clostridiales* was lower in horses with AM compared to their CoG, especially in non-surviving AM animals [35].

Clinically healthy CoG of HGA-poisoned horses are exposed to similar toxic pressure as horses affected by AM, yet they do not exhibit any clinical signs of poisoning [36,37,38]. Due to their shared environment and, therefore, exposure to the same protoxins, their blood often contains detectable levels of HGA and, in some cases, MCPA-carnitine [10,17,37,39] at levels that sometimes overlap with those observed in clinically affected AM horses [16,38]. Despite the absence of overt clinical signs, acylcarnitines profiling—which is the diagnostic and prognostic gold standard of AM [15,16]—revealed that many CoG has increased levels of acylcarnitines compared to control horses (i.e., horses free of toxin) [16]. This observation indicates the existence of subclinical cases among CoG, challenging their status as a healthy control group. Furthermore, another study on *Acer pseudoplatanus* poisoning in herbivorous species highlighted the existence of subclinical poisoning in species other than equids and hypothesised a potential role of gut morphology and intestinal microbiota in the risk of HGA intoxication [40]. Therefore, to study the potential role of microbiota in AM, it is necessary to include a control group composed of clinically healthy toxin-free horses and compare this group to both AM horses and their CoG.

As previously mentioned, some bacteria possess the enzymatic machinery necessary to metabolise HGA into MCPA. This raises the hypothesis that there could be a correlation between the host’s microbiota and blood parameters related to intoxication (such as HGA, MCPA-carnitine and the acylcarnitines profile). This hypothesis was not addressed in the study of Wimmer-Scherr et al. (2021), highlighting a second point for improvement in understanding the possible role of intestinal microbiota in AM [35].

Therefore, the aims of this study were (1) to compare the faecal microbiota of AM horses (both survivors and non-survivors) and their CoG with that of control horses (i.e., grazing horses without protoxins or toxic metabolites in their blood) and (2) to analyse the correlation between faecal microbiota and blood parameters associated with AM intoxication in horses (i.e., HGA, MCPA-carnitine and the acylcarnitines profile).

## 2. Materials and Methods

All procedures in this study adhered to both national and international guidelines on animal welfare. The Animal Ethics Committee of the University of Liege was consulted, and it was confirmed that the sampling process was part of routine veterinary practice for diagnosing or preventing AM. As a result, formal ethical approval was not required. Informed consent was obtained from horse owners prior to their inclusion in the study.

### 2.1. Horses: Study Design, Inclusion Criteria and Group Definition

Four separate groups were defined for the purpose of this study:

- Control horses (CONTROL): clinically healthy horses free of HGA and MCPA-carnitine in the blood (i.e., HGA and MCPA-carnitine levels below the limit of detection), living in a place where AM cases had previously been observed and spending at least 6 h a day at pasture;
- Cograzers (CoG): clinically healthy horses grazing in a pasture where a case of AM had been diagnosed in the previous 24 h, which had a normal physical exam and a normal dynamic examination at walk (no signs of AM or other obvious disease) at the time of sampling;
- AM survivors (AM–S): horses diagnosed with AM that were discharged from the clinic once free of clinical signs after a variable hospitalisation period;
- AM non-survivors (AM–NS): horses diagnosed with AM that died from the intoxication during hospitalisation or had to be euthanised due to significant clinical deterioration or continuous or unmanageable pain leading to a poor prognosis [41].

These last two groups (i.e., AM-S and AM-NS) were merged into a single group, referred to as ‘diseased horses’, for certain statistical analyses to facilitate comparisons with previously published data [16].

The groups AM-NS, AM-S and CoG include published data from a prospective clinical study conducted from autumn 2016 until spring 2019 [35]. The horses from the CONTROL group were prospectively sampled in autumn 2020.

The diagnosis of AM was based on (1) the algorithm proposed by van Galen et al. (2012) [1] (i.e., a compatible history and clinical signs highly suggestive of AM during spring/autumn) and on (2) the presence of HGA and MCPA-carnitine in serum, a modified acylcarnitines profile compatible with the diagnosis of AM, and elevated serum activities of creatine kinase (CK) when available. For inclusion in the present study, only horses with available data on blood levels of HGA, MCPA-carnitine, and acylcarnitines were considered [14,15,16].

### 2.2. Comparison of Faecal Microbiota Between Groups

A fresh faecal sample was collected at the time of clinical admission for horses suspected of AM. Faecal samples of CoG of these diseased horses were taken within 24 h of the first horse in the shared pasture displaying clinical signs of AM.

The centre of a faecal ball was sampled after direct collection from the rectum or from a pile of recently passed faeces (<30 min) as described by Stewart et al. (2018) [42] and was directly placed in a conservation medium (Stool DNA stabiliser, PSP^®^ Spin Stool DNA Plus Kit 00310, Invitek, Berlin, Germany) and stored at −20 °C until total bacterial DNA extraction.

#### 2.2.1. Bacterial DNA Extraction and High-Throughput Sequencing

The PSP Spin Stool DNA Plus Kit 00310 (Invitek, Berlin, Germany) was used to extract total bacterial DNA from stool samples as recommended by the manufacturer. The following primers (with Illumina overhand adapters), forward (5′-GAGAGTTTGATYMTGGCTCAG-3′), and reverse (5′-ACCOGCOGGCTGCTGGCAC-3′) were used to perform PCR amplification of the 16S rDNA V1–V3 hypervariable region and library preparation. Each PCR product was purified with the Agencourt AMPure XP bead kit (Beckman Coulter, Pasadena, CA, USA) and subjected to a second PCR round for indexing using Nextera XT index primers 1 and 2. After purification, PCR products were quantified using the Quant-IT PicoGreen (ThermoFisher Scientific; Waltham, MA, USA) and diluted to 10 ng/μL. A final quantification of each library was performed using the KAPA SYBR^®^ FAST qPCR Kit (KapaBiosystems; Wilmington, MA, USA) before normalisation, pooling and sequencing on a MiSeq sequencer using V3 reagents (Illumina; San Diego, CA, USA). Commercial Mock community positive controls using DNA from 10 defined bacterial species (ATCC MSA-1000, ATCC, Manassas, VI, USA) and negative controls (from extraction and PCR steps) were included in the sequencing run [43].

Raw amplicon sequencing libraries were submitted to the NCBI database under bioproject number PRJNA1170059.

#### 2.2.2. Sequence Analysis and 16S rDNA Profiling

Sequence read processing was performed as previously described [43] using the MOTHUR software package v1.48 [44] and the VSEARCH algorithm for chimera detection [45]. For operational taxonomic unit (OTU) generation, a clustering distance of 0.03 was used. 16S reference alignment and taxonomical assignment, from phylum to genus, were performed with MOTHUR and were based upon the SILVA database (v1.38.1) of full-length 16S rDNA sequences [46].

#### 2.2.3. Data Analysis

Subsampled datasets with 10,000 cleaned reads per sample were obtained and used to evaluate *α*-diversity and *β*-diversity using a vegan package (v 2.6-6.1) [47].

The analysis of *α*-diversity (i.e., measuring diversity within the community) and *β*-diversity (i.e., measuring diversity between communities or within the same community at different time points by considering sequence abundances or by considering only the presence–absence of sequences) are used to assess the ecology of a microbial community [48,49]. Indicators of *α*-diversity include the Chao richness index, reciprocal Simpson microbial diversity, and Simpson-derived evenness. Richness quantifies the number of species present within a community, while evenness describes how uniformly individuals are distributed among the species, highlighting the presence or dominance of certain species [50].

Differences in *α*-diversity between groups (AM-NS, AM-S, CoG, CONTROL) were evaluated with an ANOVA test followed by paired post hoc tests corrected with a two-stage linear step-up procedure of Benjamini, Krieger and Yekutieli using PRISM 10 (GraphPad Software; San Diego, CA, USA). Differences were considered significant for a *p* and *q*-value of 0.05 or less.

The *β*-diversity was analysed using vegan and vegan3d packages (v 1.3-0) [51] in R. Sample *β*-diversity was visualised with a Bray–Curtis dissimilarity matrix-based non-parametric dimensional scaling (NMDS) model. Differences between groups for the sample clustering and *β*-dispersion were assessed with analysis of variance (Adonis2), using Bray–Curtis dissimilarity matrices (Adonis2) tests, and post hoc paired tests (pairwise adonis package v 0.4.1) [52] with *p* threshold of 0.05, using R studio.

The differential abundance analysis of the genus populations of the whole system was carried out using the Aldex function (unpaired test in ALDEX2 package v1.36.0) [53]. The Monte Carlo method used in Aldex involves generating random samples from the dataset and calculating the statistics of interest for each sample. By repeating this process, an empirical estimate of the null statistical distribution is obtained—that is, the distribution assuming no significant difference between the randomly generated samples. The *p*-values can then be calculated by comparing the observed statistics with this empirical null distribution.

A differential abundance analysis was performed with the Deseq2 package in R (v1.44.0) to highlight statistical differences in population abundance between pairs of groups.

Finally, the theoretical presence of BCAT (EC 2.6.1.42) and BCKDHc (E_1_: EC 1.2.4.4–E_2_: EC 2.3.1.168.–E_3_: EC 1.8.1.4. for the three different subunits respectively) in bacterial profiles were investigated with Picrust2 tool [54]. This tool allows for theoretical metagenome function prediction based on 16S sequences. Orthologous sequences corresponding to these enzymes were identified using the Kyoto Encyclopaedia of Genes and Genomes (KEGG) database (ver. 2024-11-20, http://www.kegg.jp/kegg/, accessed on 23 November 2024). The presence of these orthologous sequences in the genus populations from our dataset was identified from known genome content present in the database. The statistical analyses and graphical representations of the number of pseudo-counts of each ortholog identified by groups of horses were performed using GraphPad Prism 10 (GraphPad Software; San Diego, CA, USA). The Kolmogorov-Smirnov test was used to assess the normality or lognormality of the data distribution, followed by a Kruskal-Wallis multiple comparisons test.

### 2.3. Correlation Between Faecal Microbiota and Blood Parameters

A blood sample was collected from each diseased horse upon admission to the clinic. Cograzers of these diseased horses were sampled in the field within 24 h after the first affected horse in the pasture showed clinical signs of AM. Blood samples were obtained via jugular venipuncture, aliquoted within one hour of collection, and stored at −80 °C until analysis. Both blood samples and faecal samples were collected simultaneously from each horse.

#### 2.3.1. Quantification of Hypoglycin A, Methylenecyclopropylacetyl-Carnitine and Acylcarnitines

The quantification of HGA was performed on serum according to a previously described methodology using aTRAQ^®^ kit (Sciex, Framingham, MA, USA) for amino acid analysis of physiological fluids [17]. Briefly, HGA was derivatised using an isotopic tag (mass *m*/*z* 121), while a second labelling reagent (mass *m*/*z* 113) allowed absolute quantification. The samples were derivatised and introduced into a TQ5500 tandem mass spectrometer (Sciex, Framingham, MA, USA) using a Prominence AR HPLC system (Shimadzu, Kyoto, Japan). The lower limit of quantification associated with this method is 0.090 μmol/L and a coefficient of variation below 8% [17].

An ultra-performance liquid chromatography combined with subsequent tandem mass spectrometry (UPLC-MS/MS) was used for MCPA-carnitine quantification with a limit of detection of approximately 0.001 nmol/L, as previously described [2].

Free carnitine and acylcarnitine profiling were quantified in serum by tandem mass spectrometry. Serum proteins were precipitated with a methanol solution with labelled internal standards. After evaporation with a nitrogen stream and derivatisation with butanolic-HCl, the samples were analysed with a TQ5500 mass spectrometer (Sciex, Framingham, MA, USA) [15,55].

#### 2.3.2. Statistical Analysis of Group Parameters and Blood Markers Associated with Intoxication

The statistical analyses and graphical representations were performed using GraphPad Prism 10 (GraphPad Software; San Diego, CA, USA). The Kolmogorov-Smirnov test was employed to assess the assumption of normal or lognormal distribution: the data were normalised through Log10 transformation if needed.

A comparison of the means of the parameters “Age” and “Sex” and the blood concentration of HGA and MCPA-carnitine was conducted by comparing groups as previously described in the literature [16].

The acylcarnitines profiling was studied by comparing the averages between groups of horses and by focusing on the recently published cutoffs isovaleryl-/2-methylbutyrylcarnitine (i.e., C5 acylcarnitine) [13,15,16].

#### 2.3.3. Statistical Analysis of the Correlation Between Faecal Microbiota and Blood Parameters

A Mantel test was performed to assess the correlation between faecal microbiota and blood parameters related to intoxication: HGA, MCPA-carnitine and a selection of acylcarnitines (i.e., C2, C4, C5, C10, C12:1, C14, C14:1, and C18:1) in serum, based on previous publications [15,16].

A multivariate analysis of the influence of selected acylcarnitines, HGA and MCPA-carnitine on microbiota distribution was performed using a distance-based redundancy analysis (dbRDA) with the vegan package in R software. This dbRDA was performed (1) to explore the interactions between the chemical variables (i.e., HGA, MCPA-carnitine and selected acylcarnitines) and the microbial composition of samples, (2) to identify the most important variables, (3) to visualise results, and (4) to statistically validate relationships between variables and sample composition. The significance of parameter influence on the dimensional model was assessed with ANOVA (vegan package). A *p*-value of 0.05 or less was considered statistically significant. The dbRDA model was illustrated with the ggord package in R (v1.1.8) [56].

## 3. Results

### 3.1. Horses

Changes in the classification of bacteria since 2021 justified a re-analysis of faecal samples from AM horses and CoG coming from Wimmer-Scherr and collaborators’ study [35]: during this process, one animal was discarded due to lack of bacterial DNA in the faecal samples to allow further analysis.

A total of 36 horses were included: 13 horses in the AM-NS, 12 horses in the AM-S, 5 horses in the CoG, and 6 horses in the CONTROL group.

The final population included a mix of different breeds: Andalusians, Belgian Warmblood Horses, Belgian Draft Horses, Friesians, Haflingers, Hanoverians, Irish Cobs, Merens, Ponies, Quarter Horses, French saddlebreds, Trotters and Zangersheides. The age and sex distribution of each group are presented in Table 1.

### 3.2. Comparison of Faecal Microbiota Between Groups

#### 3.2.1. Composition of Faecal Microbiota

Starting with 6,099,718 raw reads, 3,649,756 reads were kept after read cleaning and chimera removal. We further proceeded with 10,000 reads per sample to the taxonomic identification, leading to a table of 184,346 OTUs.

**Figure A1:**
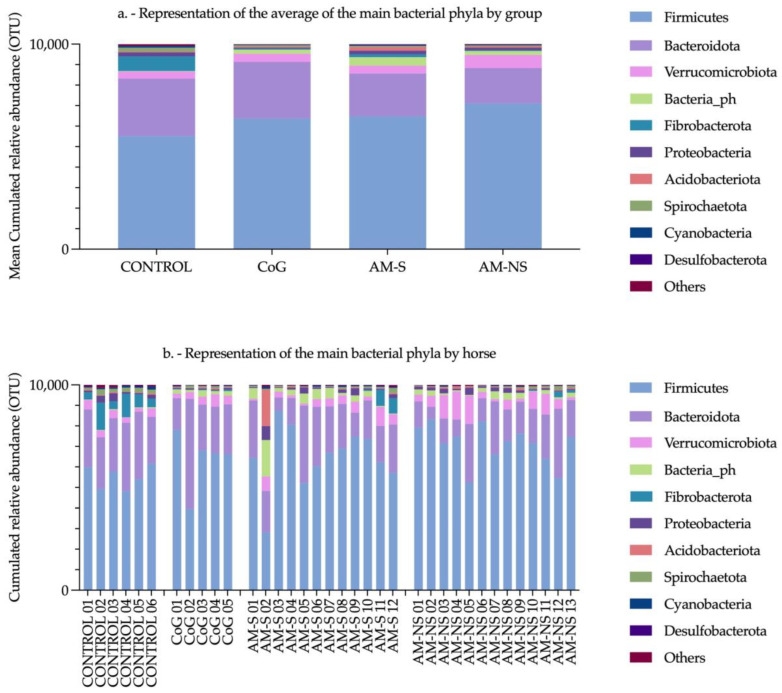
Main dominant bacterial phyla: (**a**) by group and (**b**) by horse. The horses represented are control horses (CONTROL), cograzers (CoG), survivors (AM-S) and non-survivors (AM-NS) of atypical myopathy.

**Figure A2:**
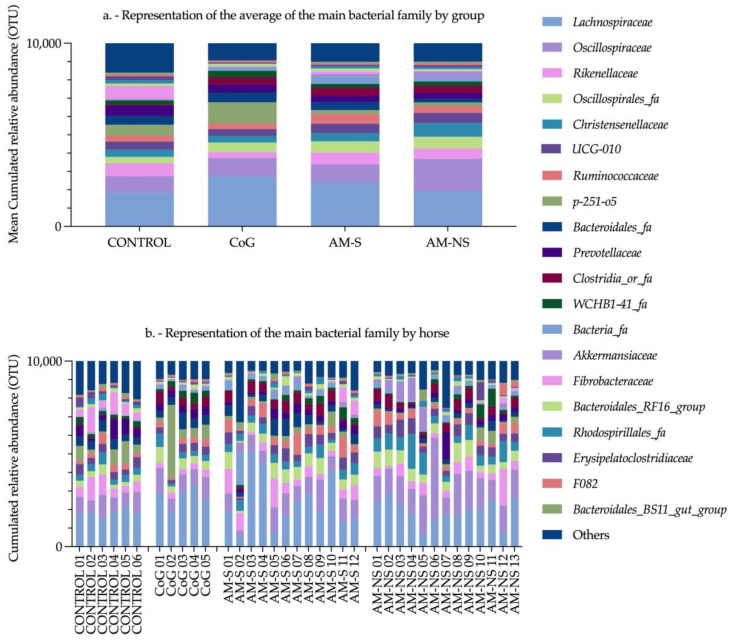
Main dominant bacterial family: (**a**) by group and (**b**) by horse. The horses represented are control horses (CONTROL), cograzers (CoG), survivors (AM-S) and non-survivors (AM-NS) of atypical myopathy.

**Figure A3:**
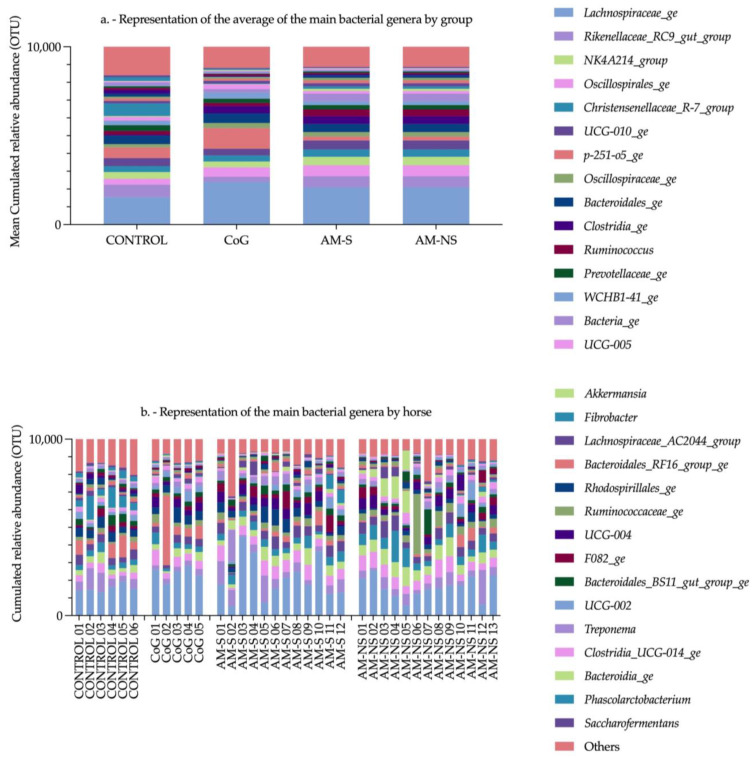
Main dominant bacterial genera: (**a**) by group and (**b**) by horse. The horses represented are control horses (CONTROL), cograzers (CoG), survivors (AM-S) and non-survivors (AM-NS) of atypical myopathy.

Among the 21 phyla identified in the faeces, the 4 most abundant defined phyla were Firmicutes, Bacteroidota, Verrucomicrobiota and Fibrobacterota. A total of 206 families and 440 genera were identified. The dominant bacterial populations for each group and individual are detailed in Appendix A. Figures show results by group and for each individual in terms of phyla (Figure A1), family (Figure A2) and genera (Figure A3). The determination of *α*- and *β*-diversities of the faecal bacterial populations were assessed at the genus level.

#### 3.2.2. *α*-Diversity Analysis

**Figure 1:**
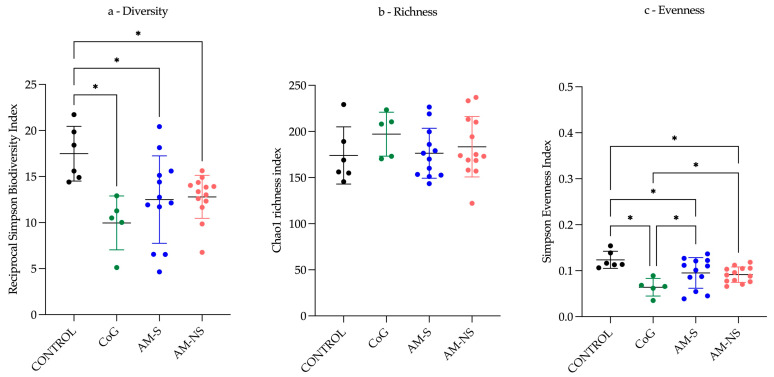
Representation of bacterial intrinsic diversity deduced from inverse Simpson index ((**a**)—Diversity), bacterial genus richness deduced from Chao1 index ((**b**)—Richness) and bacterial genus evenness deduced from Simpson index ((**c**)—Evenness). Data are scatter dot plots at the genus level for individual horses in the defined groups (control horses (CONTROL), cograzers (CoG), survivors (AM-S) and non-survivors (AM-NS) among atypical myopathy cases), with the mean and the standard deviation. The two-stage linear step-up procedure of Benjamini, Krieger and Yekutieli reveals significant differences with a *q*-value of 0.05 or less: * < 0.05.

A test of normality was performed, and a normal distribution of data was indicated. Consequently, an ANOVA and a paired test were performed (*p* = 0.05). The outcomes of Benjamini, Krieger and Yekutieli with a false discovery rate (FDR) (*q* = 0.05) indicated significant disparities among the groups concerning bacterial α-diversity (reciprocal Simpson Index) and evenness (i.e., distribution of abundances of the groups—Simpson Evenness index), though not for richness (i.e., number of taxonomic groups—chao1 richness index) (Figure 1).

Pairwise comparisons showed that α-diversity was significantly higher in CONTROL horses than in CoG (*q* < 0.01), in AM-S (*q* < 0.01), and in AM-NS (*q* < 0.05). Similarly, genus evenness was found to be (1) significantly higher in the CONTROL group vs. CoG (*q* < 0.001), AM-S (*q* < 0.05), and AM-NS (*q* < 0.05) and (2) significantly lower in the CoG group vs. AM-S (*q* < 0.05) and AM-NS (*q* < 0.05) (Table 2).

#### 3.2.3. *β*-Diversity Analysis

**Figure 2:**
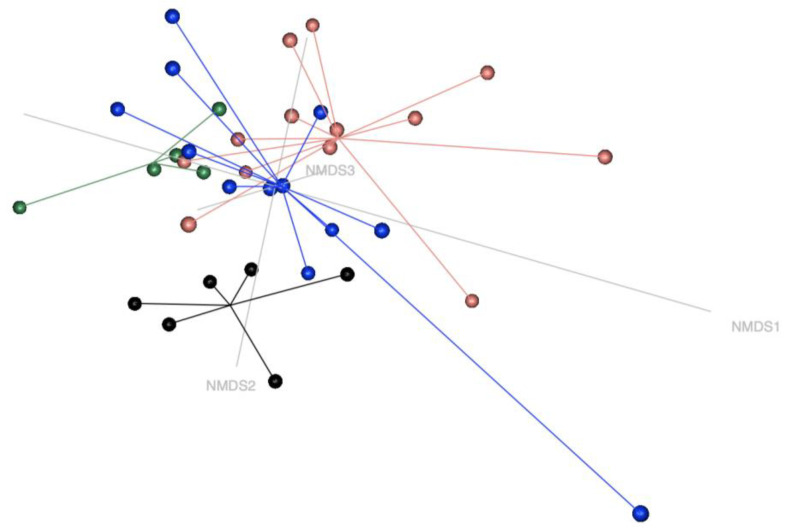
Nonmetric multidimensional scaling model (NMDS) plots in three dimensions of the horse’s faecal microbiota. Black symbols represent control horses (CONTROL), green symbols represent cograzers (CoG), blue symbols represent survivors of atypical myopathy (AM-S), and red symbols represent non-survivors (AM-NS). The model stress is 0.082.

*β*-diversity of the faecal microbial profile was visualised using a Bray-Curtix matrix-based NMDS model (k = 3, stress = 0.082) (Figure 2). Sample clustering showed that bacterial profiles were not homogenous between groups (*p* = 0.001). Results from paired tests (*p* = 0.05) showed that the microbial profile of the CONTROL group was different from the other groups, and CoG was different from AM-NS, as shown in Table 3.

#### 3.2.4. Differences in Global Faecal Microbiota Composition

The Aldex function revealed six significantly different genera in the samples studied (without the notion of group), compared with the null statistical distribution empirically determined by this aldex function: *Clostridia_ge*, *Bacteria_ge*, *Firmicutes_ge*, *Phascolarctobacterium*, *Fibrobacter* and *NK4A214_group* (Table 4).

#### 3.2.5. Differences in Faecal Microbiota Composition Between Groups

**Figure 3:**
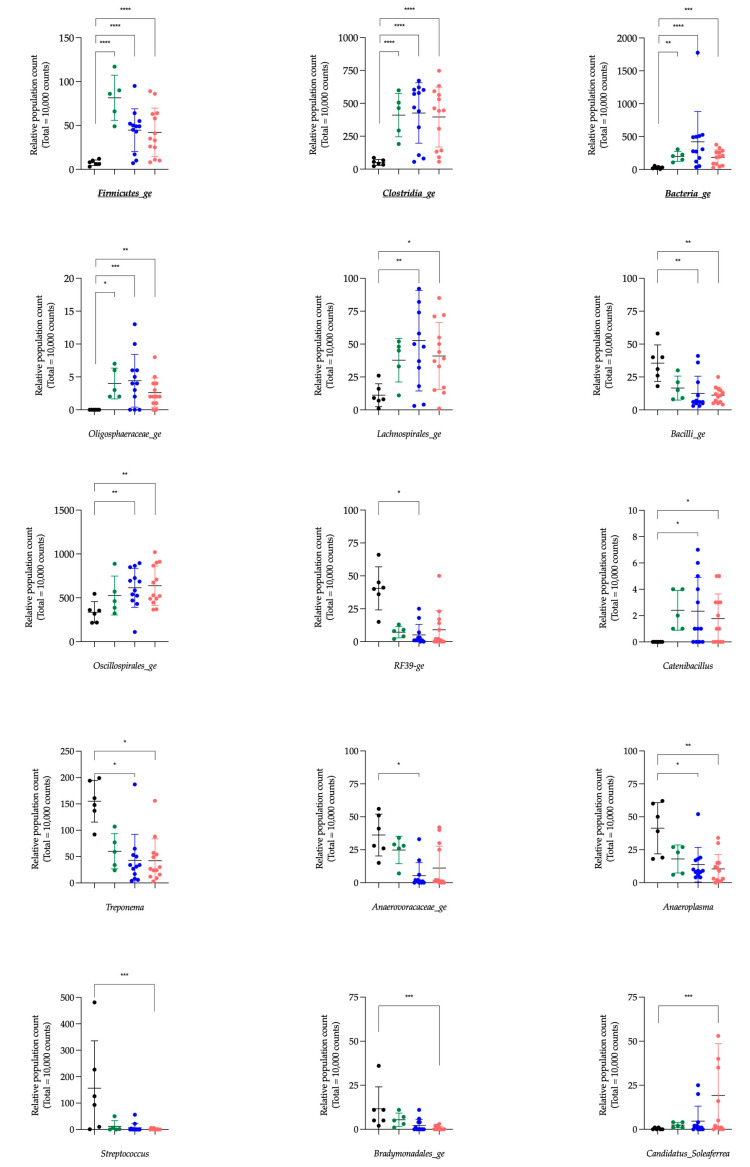

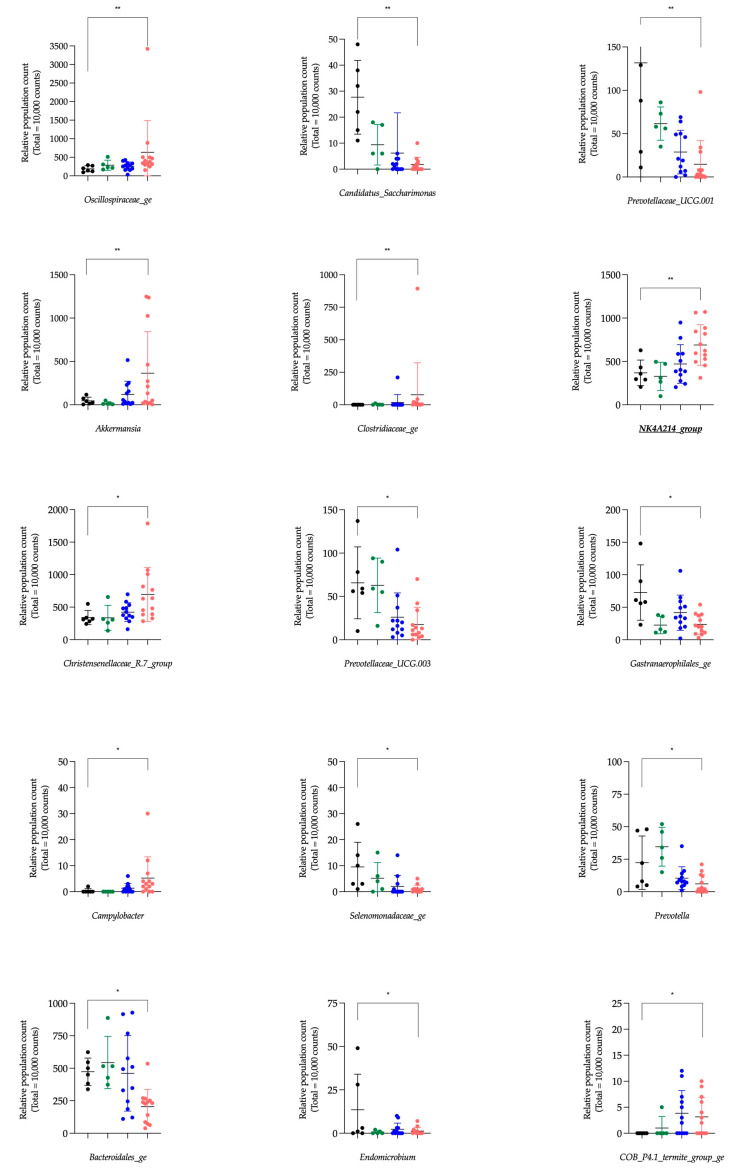

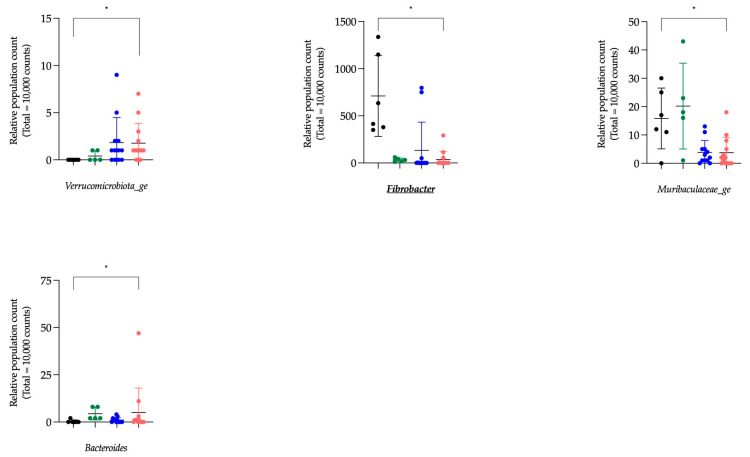
Graphical representation of relative population count (mean and standard deviation, total of 10,000 counts). Data are scatter dot plots at the genus level for individual horses in the defined groups, with black dots for control horses (CONTROL), green dots for cograzers (CoG), blue dots for survivors (AM-S) and red dots for non-survivors (AM-NS) of atypical myopathy. Genus names in bold and underlined were also highlighted by the Aldex function. Significantly different with a *p*-value of 0.05 or less: * < 0.05; ** < 0.01; *** < 0.001, **** < 0.0001.

At a genus level, the statistically significant differences in population abundance between the CONTROL group and the three other groups (CoG, AM-S and AM-NS, respectively) are represented in Table 5. From a total of 34 genera, 4 genera were statistically different between CONTROL and CoG, 12 genera were statistically different between CONTROL and AM-S, and 32 genera were statistically different between CONTROL and AM-NS. These genera are graphically represented in Figure 3.

The 4 genera statistically different between CONTROL and CoG were also different between CONTROL and AM-S and AM-NS. Interestingly, the relative abundance of these four genera was significantly lower in CONTROL vs. CoG, AM-S and AM-NS. These genera are *Firmicutes_ge*, *Clostridia_ge*, *Bacteria_ge* and *Oligosphaeraceae_ge*. Three of them (i.e., *Firmicutes_ge*, *Clostridia_ge* and *Bacteria_ge*) were also highlighted by the Aldex function. The genera *NK4A214_group* and *Fibrobacter*—also revealed by the Aldex function—presented a significantly lower and higher relative abundance between CONTROL and AM-NS, respectively.

**Table 5:** Adjusted *p*-value corresponding to comparisons between groups.

Adjusted *p*-Value	CONTROL vs. CoG	CONTROL vs. AM-S	CONTROL vs. AM-NS
*Firmicutes_ge*	****	****	****
*Clostridia_ge*	****	****	****
*Bacteria_ge*	**	****	***
*Oligosphaeraceae_ge*	*	***	**
*Lachnospirales_ge*		**	*
*Bacilli_ge*		**	**
*Oscillospirales_ge*		**	**
*RF39_ge*		*	
*Catenibacillus*		*	*
*Treponema*		*	*
*Anaerovoracaceae_ge*		*	
*Anaeroplasma*		*	**
*Streptococcus*			***
*Bradymonadales_ge*			***
*Candidatus_Soleaferrea*			***
*Oscillospiraceae_ge*			**
*Candidatus_Saccharimonas*			**
*Prevotellaceae_UCG.001*			**
*Akkermansia*			**
*Clostridiaceae_ge*			**
*NK4A214_group*			**
*Christensenellaceae_R.7_group*			*
*Prevotellaceae_UCG.003*			*
*Gastranaerophilales_ge*			*
*Campylobacter*			*
*Selenomonadaceae_ge*			*
*Prevotella*			*
*Bacteroidales_ge*			*
*Endomicrobium*			*
*COB_P4.1_termite_group_ge*			*
*Verrucomicrobiota_ge*			*
*Fibrobacter*			*
*Muribaculaceae_ge*			*
*Bacteroides*			*

Significantly different with a *p*-value of 0.05 or less: * < 0.05; ** < 0.01; *** < 0.001, **** < 0.0001. The defined groups are control horses (CONTROL), cograzers (CoG), survivors (AM-S) and non-survivors (AM-NS) of atypical myopathy. The genera that were also highlighted by the Aldex function are underlined.

#### 3.2.6. Enzymes of the Kyoto Encyclopaedia of Genes and Genomes Orthologous Pseudo-Counts Analysis

The KEGG orthologous for BCAT (EC 2.6.1.42) is K00826. For the BCKDHc, the subunit E1 presents three KEGG orthologous: K00166 for 2-oxoisovalerate dehydrogenase E1 component subunit alpha, K00167 for 2-oxoisovalerate dehydrogenase E1 component subunit beta and, K11381 for 2-oxoisovalerate dehydrogenase E1 component. The KEGG orthologous for subunits E2 and E3 are K09699 and K00382, respectively.

**Figure 4:**
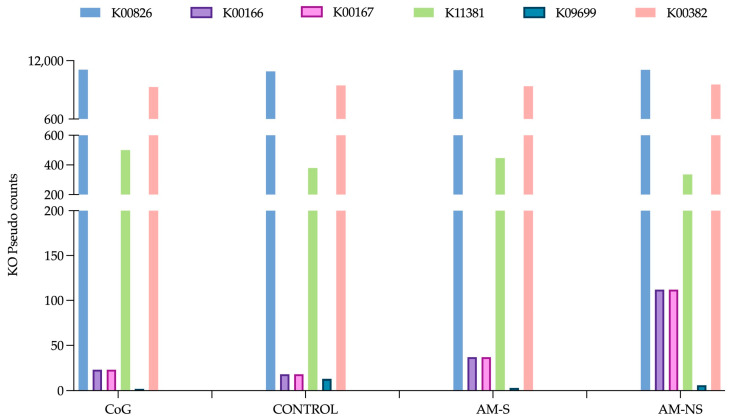
Graphical representation of each KO pseudo-counts in the defined groups: control horses (CONTROL), cograzers (CoG), survivors (AM-S) and non-survivors (AM-NS) of atypical myopathy.

Using the Picrust2 tool, the repartition of pseudo-counts of each KEGG Orthology number (KO) by groups of horses is represented in Figure 4. As observed and expected, the Kruskal-Wallis analysis of the different pseudo-counts of each K0 did not reveal any statistical differences between groups of horses (i.e., CONTROL, CoG, AM-S and AM-NS).

The general analysis of the presence of KO in each OTU indicated that (1) K00826 (i.e., BCAT) was mainly represented in 99.18% of total OTUs, (2) K00382 (i.e., BCKDHc—E3) was represented in 70.13% of total OTUs and, (3) BCKDHc E1 and E2 subunits were the main limiting subunits. The latter were poorly represented within the entire 16S rDNA (0.35%, 0.35%, 3.28% and 0.08% for K00166, K00167, K11381 and K09699, respectively), with only four OTUs possessing all the enzymes, and these same four OTUs were the only ones to possess the entire E1 and E2 subunits. Moreover, the relative abundance of these four OTUs was low. The identification of the genus of these four OTUs was *Alphaproteobacteria_ge*, *Rhizobiaceae_ge*, *Sphingomonas*, and *Sphingomonadaceae_ge*.

Among the bacterial genera highlighted by Aldex and/or Deseq2 functions, the mostly represented are K00826 (i.e., BCAT) and K00382 (i.e., BCKDHc—E3), which agrees with the general analysis (Table 6). Moreover, many of the genera analysed only presented these two orthologs. *Phascolarctobacterium* did not present any pseudo-count for any KO number like some OTUs from some genera (i.e., *Anaerovoracaceae_ge*, *Bacilli_ge*, *Bacteria_ge*, *Bacteroidales_ge*, *Christensenellaceae_R.7_group*, *Clostridia_ge*, *Firmicutes_ge*, *Lachnospirales_ge*, *Oscillospirales_ge*). None of the genera identified possessed all the orthologs. Nevertheless, some OTUs from the genera *Bacilli_ge* and *Bacteria_ge* presented 5/6 orthologous sequences with the same missing K11381 (i.e., 2-oxoisovalerate dehydrogenase E1 component). Some OTUs from the genera *Akkermansia*, *Bacteria_ge* and *Verrucomicrobiota_ge* owned 4/6 orthologous sequences, with K11381 and K09699 missing (i.e., BCKDHc E2 subunit). Finally, one OTU from *Verrucomicrobiota_ge* and *Muribaculaceae_ge*, three OTUs from *Prevotella*, and several OTUs from *Bacteria_ge*, *Bacteroidales_ge*, *Bacteroides*, *Christensenellaceae_R.7_group*, *Clostridia_ge*, *COB_P4.1_termite_group_ge*, *Firmicutes_ge*, *NK4A214_group*, *Oscillospiraceae_ge*, *Oscillospirales_ge*, *Prevotellaceae_UCG-001*, *Prevotellaceae_UCG-003* presented the same 3/6 orthologous sequences (i.e., K00826, K11381 and, K00382).

### 3.3. Correlation Between Faecal Microbiota and Blood Parameters

#### 3.3.1. Group Parameters and Blood Markers Associated with Intoxication

The average blood concentrations of HGA, MCPA-carnitine and selected acylcarnitines, as well as their standard deviation for each group, are referenced in Table A1 and Table A2.

Statistical comparison of the “Age” parameter between (1) CoG vs. diseased horses, (2) AM-S vs. AM-NS, (3) CONTROL vs. CoG and (4) CONTROL vs. diseased horses did not reveal any significant difference in mean age (Mann-Whitney test, *p* ≥ 0.05) or in the age distribution (Kolmogorov-Smirnov Z test, *p* ≥ 0.05).

Fisher’s exact test used to study contingency about parameter “Sex” and parameter “Health Status” or “Final Outcome of the disease horses” did not reveal any significant difference (*p* ≥ 0.05) for both comparisons.

**Figure A4:**
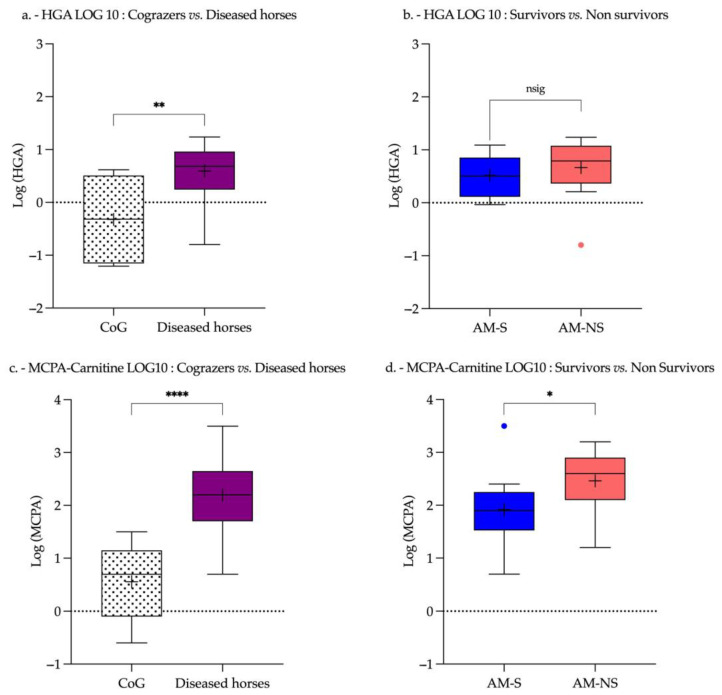
Serum hypoglycin A (HGA) (**a**,**b**) and methylenecyclopropylacetic-carnitine (MCPA-carnitine) (**c**,**d**) concentrations. Boxes range from the 25th to 75th percentiles. Means are represented by horizontal lines and medians by a cross. Box plot whiskers were established using the Tukey method. The groups are control horses (CONTROL), cograzers (CoG), survivors (AM-S) and non-survivors (AM-NS) of atypical myopathy and diseased horses. Significantly different with a *p*-value of 0.05 or less: * < 0.05; ** < 0.01; **** < 0.0001, Not significant “nsig”.

The mean serum concentration of HGA was significantly lower in CoG vs. diseased horses (unpaired *t*-test, *p* < 0.001), but the difference was not significant for the comparison AM-S vs. AM-NS (unpaired *t*-test, *p* ≥ 0.05) (Figure A4).

When comparing the mean serum concentration of MCPA-carnitine, diseased horses presented values significantly higher than CoG (unpaired *t*-test, *p* < 0.0001). Similarly, the mean serum concentration of MCPA-carnitine was significantly higher in AM-NS vs. AM-S (unpaired *t*-test, *p* < 0.05) (Figure A4).

**Figure 5:**
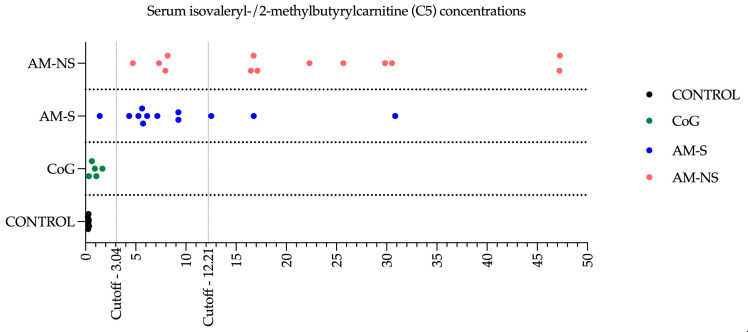
Serum isovaleryl-/2-methylbutyrylcarnitine (C5) concentrations. The groups are control horses (CONTROL), cograzers (CoG), survivors (AM-S) and non-survivors (AM-NS) of atypical myopathy.

The mean serum concentration of selected acylcarnitines in diseased horses was found to exceed the 99th percentile of the CONTROL group values. Additionally, the C5-carnitine concentration of each horse was analysed, according to what’s been proposed by Renaud et al. (2024), to classify horses into the appropriate groups. A cutoff value of 3.04 μmol/L of C5 was described: above this cutoff, 92% of horses are diseased horses, and below this cutoff, 97% of horses are CoG. A second C5 cutoff is described with a value of 12.21 μmol/L: above this cutoff, 76% of diseased horses are likely to die, and below this cutoff, 81% of diseased horses are likely to survive [16]. In this study, only one AM-S horse (i.e., AM-S 04) presented a suspect profiling with a concentration of C5 below the cutoff of 3.04 μmol/L of clinically affected AM cases. Three AM-affected horses survived despite a poor prognosis based on C5 (Figure 5), and four diseased horses died while having a C5 level, suggestive of a positive outcome.

#### 3.3.2. Correlation Between Faecal Microbiota and Serum Concentration of Hypoglycin A, Methylenecyclopropylacetyl-Carnitine and Acylcarnitines

The Mantel test revealed a significant correlation (r = 0.2351, *p* = 0.031) between the matrix of microbiota and the matrix of blood parameters, suggesting that this correlation is unlikely due to chance.

A first dbRDA model was performed: the most important variables identified were MCPA-carnitine (*p* = 0.004), C2 (*p* = 0.089), C10 (*p* = 0.099) and C14:1 (*p* = 0.079).

**Figure 6:**
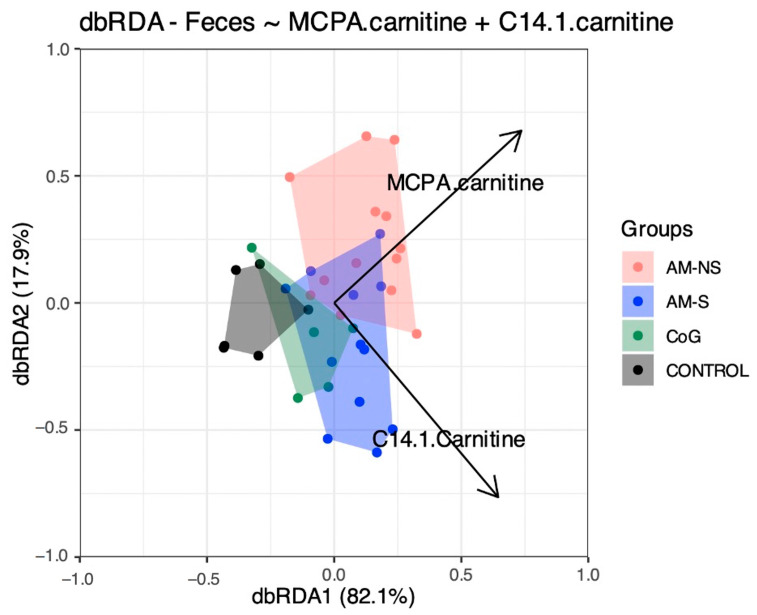
Illustration of the dbRDA model. The horizontal dbRDA1 axis explains 82.1%, and the vertical dbRDA2 axis explains 17.9% of the repartition of the different groups: control horses (CONTROL), cograzers (CoG), survivors (AM-S) and non-survivors (AM-NS) of atypical myopathy.

A series of dbRDA models were further built iteratively by sequentially removing the significant chemical variables identified previously. This allowed us to select the most important variables to explain the variation in microbial composition between groups: these variables were MCPA-carnitine (*p* < 0.01) and C14:1 (*p* < 0.01), resulting in a final dbRDA model with two constrained dimensions (Figure 6).

## 4. Discussion

The present study reveals that (1) faecal microbiota differs between CONTROL horses (i.e., toxin-free horses) and horses suffering from both subclinical (CoG) and clinical (AM-S and AM-NS) *Acer pseudoplatanus* intoxication and (2) the blood concentrations of MCPA-carnitine and C14:1 (i.e., tetradecenoylcarnitine, a long-chain acylcarnitine) significantly correlate with the variation in faecal microbial composition observed between the different groups of horses.

Microbiota statistical analyses were performed at the genus level to detect differences at the highest possible taxonomic resolution. This approach allows for a more accurate identification of the microbial populations influencing the system under investigation using the most up-to-date bacterial taxonomy. Unfortunately, recent updates in bacterial taxonomy since the publication of Wimmer-Scherr et al. (2021) have complicated a direct comparison of the results between the two studies [35]. Nevertheless, the findings of this new study will facilitate future comparisons with other research.

Regarding *α*-diversity, the CONTROL group presents the highest *α*-diversity and exhibits a more uniform distribution of populations compared to the three other groups, with an evenness index closer to one. The CoG group presents a lower *α*-diversity and an evenness index close to zero, indicating the emergence of certain populations more abundant than others. “Dysbiosis” is defined as the loss of central mutualistic relationship among microbiota members, metabolic products, and the host immune system [57]. Consequently, it is not possible to characterise the observed changes as dysbiosis only based on *α*-diversity indicators.

The Aldex function identified six genera having a significant impact in the global system (i.e., *Clostridia_ge*, *Bacteria_ge*, *Firmicutes_ge*, *Phascolarctobacterium*, *Fibrobacter* and *NK4A214_group*) compared to the null statistical distribution empirically determined by this function. The paired tests (Deseq2) compared bacterial genera between the CONTROL group and the three other groups and revealed 34 significantly different genera. Among these bacterial genera, five were also highlighted by the Aldex function. Indeed, *Clostridia_ge*, *Bacteria_ge*, and *Firmicutes_ge* presented a significantly lower relative abundance in the CONTROL group compared to CoG, AM-S and AM-NS groups, and *NK4A214_group* and *Fibrobacter* in the CONTROL group exhibit a significantly lower and higher relative abundance, respectively, compared to AM-NS horses.

The genera *Clostridia_ge*, *Bacteria_ge*, and *Firmicutes_ge* are composed of bacteria with features belonging to the *Clostridia* class, Bacteria kingdom or Firmicutes phyla, respectively, without the possibility of further characterisation. The genus *Fibrobacter* is a major and highly specialised cellulolytic bacterial genus and is detected in the intestinal tract of several herbivorous animals, including horses [58,59]. Cellulose is not digested and not absorbed in the mammal’s gut, so cellulolytic bacteria play a vital role by providing energy to the host via the metabolism of cellulose into short-chain fatty acids (SCFAs). The SCFAs have beneficial effects on colonocytes, intestinal membrane integrity and local intestinal immunity [60,61]. The toxic metabolites of protoxins (i.e., MCPA-CoA and MCPF-CoA) inhibit the *β*-oxidation of fatty acids with subsequent decreased mitochondrial respiration and uncoupled phosphorylation [5,12,13]. Therefore, lipids can no longer be used as an energy substrate, but the glycolytic pathway is preserved [14,62,63]. Consequently, it is generally recommended to give a complete mix of grains or glucose in another form to horses affected by AM [64]. Although the appetite of diseased horses is preserved, the clinical picture (weakness, recumbency, depression, stiffness, etc. [1,36,65]) can make an inadequate ingestion of food. It is hypothesised that the inhibition of *β*-oxidation by toxic metabolites alters the overall energy metabolism of the host and the intestinal environment, potentially leading to conditions that are less favourable for the growth and activity of cellulolytic bacteria, thus explaining the observed decrease in the relative abundance of this genus from CONTROL horses to intoxicated horses (from CoG to AM-NS).

The genus *NK4A214_group* is associated with the degradation of structural carbohydrates, such as cellulose and hemicellulose, other less complex polysaccharide substrates and the production of butyrate (i.e., SCFAs) [66,67]. The relative increase in this genus from CONTROL horses to intoxicated horses, as previously reported [35], could be linked to the preservation of the glycolytic pathway and its ability to deal with less complex polysaccharides, as mentioned above. This preservation may provide a more favourable environment for the *NK4A214_group* by maintaining substrate availability for carbohydrate fermentation despite the metabolic disruptions caused by toxic exposure.

Other clearly identified genera, such as *Akkermansia*, *Bacteroides*, *Prevotella,* and *Treponema,* might be considered interesting in the context of AM. The genus *Akkermansia* attracts the attention of scientists for its anti-inflammatory and anti-obesity effects in mice and men by reducing insulin resistance, glucose intolerance, and gut permeability. One of the distinguishing features of *Akkermansia* is its ability to (1) promote the renewal and thickening of the mucin layer, which reduces intestinal permeability, and (2) degrade intestinal mucin glycoproteins and to use them as a source of carbon and nitrogen when dietary fibres are defective; this process leads to an increase of the intestinal permeability and to the production of SCFAs [68,69,70,71,72]. This ability to use mucin as a source of carbon and nitrogen may give *Akkermansia* an advantage when facing disruptions in host energy metabolism in the context of AM, despite the potential repercussions on intestinal microbiota. On the other hand, the resulting increase in intestinal permeability represents a disadvantage, as this could increase the absorption of HGA and other toxins present in the digestive tract and into the bloodstream. Lastly, the resulting production of SCFAs—which account for 65% of the horse’s energy production [73,74]—cannot be used as an energy source by the horse. Indeed, in the case of AM, the muscle cannot use them effectively as energy substrates following the inhibition of acyl-CoA dehydrogenases [5,12,75,76,77].

The genus *Bacteroides*, which increases in AM-NS compared to CONTROL horses, is known for its ability to metabolise polysaccharides and oligosaccharides such as starch [78,79], which could make sense in the framework of the modification of the metabolic pathways available in horses affected by AM. This genus has interesting features to thrive in the gut as (1) complex systems to sense and adapt to available nutrients, (2) systems to get rid of toxic substances, and (3) the ability to control the environment by interacting with the host’s immune system, which is an opportunity to control of other pathogens considered as competitors by *Bacteroides* [78]. Moreover, the relative abundance of *Prevotella* is inversely correlated with that of the *Bacteroides* in human gut microbiota [80], and this is what is observed in the faecal microbiota analysis between CONTROL and AM-NS horses.

The genus *Treponema* is associated with the degradation of structural carbohydrates and correlates positively with different dietary fibres in pigs and horses [66,81]. In this study, *Treponema* was present in faecal microbiota in CONTROL horses and significantly decreased in AM-NS, which could also be explained by the modification of energetic metabolism in AM horses.

The explanations provided remain hypothetical. At this stage, it is not possible to draw definitive conclusions about the role of these bacterial populations in the context of AM based solely on relative abundance. By definition, the analysis of relative abundance reveals populations that are increasing and others that are decreasing relative to each other. This phenomenon is illustrated by the inverse correlation observed between *Prevotella* and *Bacteroides* in humans [80]. This illustration of the ecological niche can also explain variations in populations of genera like *Campylobacter*, *Endomicrobium*, and *Streptococcus*. It is important to remain cautious when interpreting increases or decreases in bacterial populations expressed in relative abundance values: only an absolute quantification of these populations would make it possible to truly quantify the increases and decreases (for example, via RT-PCR). Furthermore, the bacteria present in the gut microbiota are also influenced by the host’s metabolism. As a result, observations made in vivo, at the level of the faeces, may not necessarily reflect changes in the microbiota itself following exposure to the protoxins studied. Finally, it is possible that bacteria identified as significantly different between groups are involved in host-related metabolic functions rather than in the direct metabolism of HGA. The discovery of the subclinical character of metabolic processes in horses that have ingested toxins involved in AM [16] suggests that the gut microbiota also has time to modify before the appearance of the clinical phase, which remains acute.

The enzymes responsible for HGA metabolism (i.e., BCAT and BCKDHc) are involved in the catabolism of BCAAs in bacteria [82], leading to the hypothesis that bacteria can transform HGA into toxic metabolic compounds. In mammals, the BCAT exists in two isoenzymes: a mitochondrial (BCATm) ubiquitously present (mainly in skeletal muscle, brain, kidney, and intestine in humans), and a cytosolic (BCATc) isoenzyme, mainly presents in the brain in humans and rats, and in ovary and placenta in rats [83,84,85,86,87,88,89,90]. In contrast to the mitochondrial and cytosolic isoenzymes found in higher eukaryotes, a single form of BCAT is ubiquitously expressed in bacteria. Moreover, this BCAT is involved in the final step of anabolism and in the first step of catabolism of BCAAs, which explains that many bacterial species possess this BCAT. Finally, bacterial BCAT is also distinguished from eukaryotes by broad substrate specificity [91,92,93]. The mammals BCKDHc is a multienzyme complex of three separate subunits located on the inner surface of the inner mitochondrial membrane [89,94]. The bacterial BCKDHc seems to mainly consist of three subunits, as described for mammalian species [82,95] and is also found in several bacterial species [25,27,28,31,96,97]. Among the bacterial genera highlighted by Aldex function and/or Deseq2 analysis, some of them have in their known genome content one or more copies of the orthologous sequences encoding for the BCAT and/or subunits of BCKDHc (i.e., enzymes involved in the metabolisation of HGA). However, no bacterial genus appears to possess all the expected enzymes, and consequently, the question of the ability of these genera to completely transform HGA in MCPA-linked to carnitine or glycine arises. Interestingly, (1) a pattern of distribution can be observed with the same ortholog missing in groups of genera owning 5/6, 4/6 or 3/6 orthologous sequences and (2) if we compare the pattern of distribution of genera identified in the scientific literature as having the BCKDHc (i.e., *Bacillus*, *Pseudomonas*, *Staphylococcus* [24,25,26,28,29,30,31,96], this pattern is exactly the same as the pattern encountered for the genera having 5/6 orthologous sequences with only the K11381 missing (i.e., 2-oxoisovalerate dehydrogenase E1 component). Unfortunately, these OTUs presented this interesting pattern belonging to the non-clearly identified genera *Bacilli_ge* and *Bacteria_ge.* Indeed, the identification of these OTUs is stopped at the *Bacilli* class and Bacteria kingdom, respectively, preventing further possible characterisation and explanations. Nevertheless, *Bacteria_ge* was also highlighted by Aldex function (i.e., having a significant impact on the global system) and Deseq2 function (i.e., being significantly higher in CoG, AM-S and AM-NS horses vs. CONTROL horses). It will be valuable to investigate these specific OTUs from *Bacteria_ge* and compare them across groups to determine whether these specific OTUs are significantly different. The *Bacilli_ge* was highlighted by the Deseq2 function and was significantly different between CONTROL and AM-S and AM-NS but in a decreasing way from CONTROL to AM-NS: the opposite of the *Bacteria_ge*. This last fact seems to be contradictory (i.e., having the machinery to metabolise HGA but presenting an opposite evolution between the groups). However, some bacteria might possess analogous (non-orthologous) enzymes capable of performing similar functions.

The “Age” parameter did not seem significant in the horse population of the present study, contrary to recent findings in the scientific literature, which reported that diseased horses were either younger than 2 years or older than 10 years, while 90% of CoG were between 2 and 10 years of age [16]. In our study, there was an age overlap between groups. Regarding the “Sex” parameter, Renaud and collaborators found that, among horses exposed to the protoxins, geldings were less susceptible to developing AM compared to intact males and females [16]. However, in our study, neither “Sex” nor the parameters “Health Status” or “Final Outcome” were significantly associated. This may be explained by the lower number of individuals in our study as compared to the above-mentioned one.

As found previously [16], the mean serum concentrations of HGA and MCPA-carnitine were significantly different when comparing CoG and diseased horses. Among the latter, only MCPA-carnitine could differentiate AM-S from AM-NS. Interestingly, MCPA-carnitine was identified as one of the most important blood variables to explain the variation in microbial composition between groups and seemed to correlate better with AM-NS vs. AM-S.

Acylcarnitines are esters formed by the conjugation of acyl groups (notably fatty acids) with carnitine. They are typically divided into four groups: short-chain (C2–C5), medium-chain (C6–C12), long-chain (C13–C20) and very-long-chain (>C21) acylcarnitines. The primary biological function of acylcarnitines is to facilitate the transport of acyl groups from the cytosol into the mitochondrial matrix for *β*-oxidation, which in turn contributes to cellular energy production. Atypical myopathy is characterised by a severe alteration of the serum acylcarnitines profile with an increase in nearly all acylcarnitines, regardless of their chain length [4,11,13,14,15,16,63,98,99]. In the present study, acylcarnitines profiling revealed significant changes comparable to those previously described in the literature. Moreover, C5-carnitine has been highlighted as the best candidate for helping in both the diagnosis and prognosis of the disease. According to the model described by Renaud et al. (2024), a serum concentration of C5-carnitine lower than 3.04 μmol/L would make it possible to identify a diseased horse vs. a cograzer in more than 90% of cases. A second threshold at 12.21 µmol/L of C5-carnitine would identify an animal likely to die (i.e., negative predictive value) in 76% of cases and a surviving animal (i.e., positive predictive value) in 81% of cases [16]. In this study, the analysis of the C5 concentration identified one horse in the AM-S group (i.e., AM-S 04) with a profile similar to that of a CoG, as well as four other cases with prognostic survival estimates that differed from the real outcome: nevertheless, these results are consistent with the percentages announced in the literature. Of the four diseased horses that ultimately died despite having C5 levels indicative of a positive outcome, it should be noted that 3 of them were euthanised following the worsening of clinical signs despite the intensive treatment put in place, and the last was also euthanised, but the reason (i.e., medical or financial constraints) was not clearly identified. Moreover, to the authors’ knowledge, none of the CoG developed symptoms of AM during the sampling season, supporting the results obtained from the analysis of these horses’ blood concentration of C5 carnitine. Rapid access to C5 assay results remains a significant challenge, preventing clinicians from promptly updating prognoses in cases characterised by severe clinical signs.

Lastly, the C14:1 was identified as one important blood variable to explain the variation in faecal microbiota between groups. The elevated C14:1 level in neonates may indicate a very-long-chain acyl-coenzyme A dehydrogenase deficiency known under the following abbreviation VLCADD, an autosomal recessive disease [100]. In horses, AM is recognised as an acquired multiple acyl-CoA dehydrogenase deficiency [14,63,98]. The long-chain carnitine C14:1 concentration was above the reference range in AM horses [3,13], though serum concentrations showed no significant difference between surviving and deceased horses [15]. In a recent study involving a larger group of AM-affected horses, C14:1 was also identified as one of the variables with the most significant impact on distinguishing groups (comparable to those used to compare faecal microbiota in this paper), further corroborating the results presented here. However, it did not prove to be a reliable diagnostic or prognostic indicator [16]. In humans, tetradecenoylcarnitine (i.e., C14:1), as other long-chain acylcarnitines, plays a role in insulin resistance and in the development of cardiovascular diseases [101]. Most horses affected by AM show elevated plasma concentrations of cardiac troponin I, a specific biomarker of myocardial injury [36,102], and some horses exhibit specific alterations in electrocardiogram (ECG) recordings and cardiac ultrasound examination similar to those observed in mice and humans with VLCADD [103]. Its role in muscle insulin resistance [104] might contribute to explaining the hyperglycaemia observed in AM horses.

In equids, individual variations in faecal microbiota are influenced by factors such as nutrition, management practices, seasonal variation, medications, animal-related factors, pathological conditions, and stress-related factors [105]. To reduce these individual variations, horses included in the present study were all pasturing for a minimum of 6 h per day during a high-risk season for AM (i.e., autumn and spring), including the CONTROL horses that were sampled in November 2020, as 94% of “autumnal” cases occurred between October and December [106]. Seasonal variation and associated weather conditions are known to influence gut microbiota composition in horses [107,108,109]. This seasonal effect could be attributed to changes in the composition of environmental bacteria (e.g., soil and grass/haylage microbiota) or in the nutrient composition of pasture which are in turn influenced by climatic conditions [107,109,110]. In this study, AM-S and AM-NS horses were sampled in autumn and spring, while CoG and CONTROL horses were sampled in autumn. However, seasonal variations between winter and summer are reported to have only a minor impact on faecal microbiota, accounting for 2.8% of the variation, according to Theelen et al. (2021) [108]. Similarly, the variations between autumn and spring in the present study could also be considered minor. It is important to note that the year of sampling differed between groups (2016, 2017, and 2018 for clinical cases of AM, 2018 for selected CoG, and 2020 for CONTROL horses), which may introduce potential biases. To minimise the impact of this temporal variation, samples were collected under a strict protocol, stored immediately in a conservative medium, and frozen at −20 °C until analysis [42,111,112]. Moreover, analyses were conducted on a regular basis over the years to ensure that samples did not remain frozen for extended periods. Finally, sampling was also standardised using a protocol described by Stewart et al. (2018) [42], further minimising potential sampling bias.

There are some limitations in this study. Some of them are already described by Wimmer-Scherr et al. (2021): the potential bias introduced by euthanasia for ethical reasons based on the severity of the clinical signs in the AM-NS group as well as the possible medications received by some horses prior to referral to the clinic [35]. The method used for microbiota assessment also presents inherent limitations, potentially favouring or underestimating certain bacterial taxa due to the lack of absolute quantification and selection bias during the process (e.g., DNA extraction, primer selection, PCR amplification, and bioinformatics parameters) [113].

When a modification of the intestinal microbiota is associated with a pathology, the question arises whether this modification reflects the state of health of the host or influences the host’s health. Recently, Renaud et al. (2022) suggested that protoxins may be transformed by rumen microbiota, particularly in species with a long retention time, which would protect these species from developing AM-clinical signs. Indeed, the hypothesis proposed is that having a proximal fermentation compartment located before the absorption site of amino acids might be protective while having a distal fermentation compartment located after the absorption site of protoxins—as is the case in horses—might make the species more sensitive to poisoning [40]. This hypothesis can be partly explained by the fact that (1) certain bacteria can metabolise peptides and amino acids, and the protoxins are non-proteinogenic amino acids [114,115], and (2) some bacteria possess the enzymes involved in the metabolism of BCAAs [6,114], HGA, and MCPrG [6].

The identification of bacteria within the equine microbiota that can metabolise HGA and MCPrG into their corresponding toxic metabolites or potentially degrade these protoxins could be valuable in understanding species sensitivity. Moreover, this could contribute to a broader comprehension of intoxication and potentially aid in the discovery of molecules and/or bacteria capable of preventing this intoxication. To study the intestinal microbiota in this context, the use of alternative models, such as in vitro dynamic (i.e., SHIME^®^ or static fermentation models (i.e., batch), makes sense [116,117,118]. In addition to aligning with the three R’s—replacement, reduction, and refinement—this type of model would eliminate the direct influence of host metabolism on the microbiota by using faeces of healthy donors (i.e., CONTROL horses) and by adding the studied challenge (for example, the addition of HGA). As such, the changes observed in the digestive microbiota would be directly linked to the challenge applied to the system: the addition of protoxins in the AM model. The identification of bacteria that could play a role in HGA and/or MCPrG poisoning would then be easier. Another benefit of this kind of in vitro dynamic or static fermentation model is that it can offer opportunities for studying treatments by also adding targeted molecules. Finally, another possibility would be to use metagenomic shotgun analysis. This analysis makes it possible to describe the taxonomic composition of a community of organisms and its diversity, as well as its genes and, therefore, its functional capacities.

## 5. Conclusions

For the first time, a correlation has been observed between blood parameters and the intestinal microbiota of horses suffering from AM. At this stage, it remains to be determined whether these changes result directly from the protoxins’ effect on the microbiota, the metabolism of protoxins by bacteria, and/or the host’s pathological state. Further investigation is necessary to elucidate the underlying mechanisms and determine the specific contributions of each factor. Understanding these relationships could deepen our knowledge of the role of the intestinal microbiota in AM and open the path to potential therapeutic strategies.

## Figures and Tables

**Table 1:** Demographic data of horses classified by group.

	CONTROL	CoG	AM-S	AM-NS	Diseased Horses
Number of horses	6	5	12	13	25
Age mean ± SD (Years)	13.3 ± 8.5	7.1 ± 7.5	6.5 ± 7.7	5.8 ± 4.7	6.1 ± 6.2
Age minimum (Years)	4.0	1.5	0.4	0.5	0.4
Age maximum (Years)	23	17	25	16	25
Age CI [LLCI-ULCI]	[6.5–20.1]	[0.5–13.7]	[2.1–10.9]	[3.3–8.3]	[3.7–8.5]
Ratio of					
entire male	33%	40%	33%	15%	24%
gelding	33%	20%	33%	23%	48%
female	33%	40%	33%	62%	28%

SD = Standard deviation, CI = Confidence interval, LLCI = Lower Limit of Confidence Interval, ULCI = Upper Limit of Confidence Interval. The groups represented are control horses (CONTROL), cograzers (CoG), survivors (AM-S) and non-survivors (AM-NS) of atypical myopathy, as well as all diseased horses (AM-S + AM-NS).

**Table 2:** *α*-diversity: *p*-value for pairwise comparisons and *q*-value for the two-stage linear step-up procedure of Benjamini, Krieger and Yekutieli.

DIVERSITY			*p*-Value	*q*-Value
**CONTROL**	**vs.**	**CoG**	**	**
**CONTROL**	**vs.**	**AM-S**	**	**
**CONTROL**	**vs.**	**AM-NS**	*	*
CoG	vs.	AM-S	nsig	nsig
CoG	vs.	AM-NS	nsig	nsig
AM-S	vs.	AM-NS	nsig	nsig
**EVENNESS**			***p*-Value**	***q*-Value**
**CONTROL**	**vs.**	**CoG**	***	***
**CONTROL**	**vs.**	**AM-S**	*	*
**CONTROL**	**vs.**	**AM-NS**	*	*
**CoG**	**vs.**	**AM-S**	*	*
**CoG**	**vs.**	**AM-NS**	*	*
AM-S	vs.	AM-NS	nsig	nsig

Significantly different with a *p*- or *q*-value of 0.05 or less: * < 0.05; ** < 0.01; *** < 0.001, nsig: Not significant. The defined groups are control horses (CONTROL), cograzers (CoG), survivors (AM-S) and non-survivors (AM-NS) of atypical myopathy.

**Table 3:** *β*-diversity: *p*-value adjusted for pairwise comparisons.

	Pairs		*p*-Value Adjusted
**CONTROL**	**vs.**	**CoG**	*
**CONTROL**	**vs.**	**AM-S**	*
**CONTROL**	**vs.**	**AM-NS**	**
CoG	vs.	AM-S	nsig
**CoG**	**vs.**	**AM-NS**	*
AM-S	vs.	AM-NS	nsig

Significantly different with a *p*-value of 0.05 or less: * < 0.05; ** < 0.01; nsig: Not significant. The defined groups are control horses (CONTROL), cograzers (CoG), survivors (AM-S) and non-survivors (AM-NS) of atypical myopathy.

**Table 4:** Aldex function: adjusted *p*-value of selected genera.

Genera	*p*-Value Adjusted
*Clostridia_ge*	**
*Bacteria_ge*	**
*Firmicutes_ge*	**
*Phascolarctobacterium*	*
*Fibrobacter*	*
*NK4A214_group*	*

Significantly different with a *p*-value of 0.05 or less: * < 0.05; ** < 0.01.

**Table 6:** The presence of orthologous sequences in bacterial genera is of interest.

Orthologs Number KEGG	K00826	K00166	K00167	K11381	K09699	K00382
*Akkermansia*	✓	✓	✓			✓
	✓					✓
*Anaeroplasma*	✓					✓
						✓
*Anaerovoracaceae_ge*	✓					✓
*Bacilli_ge*	✓	✓	✓		✓	✓
	✓					✓
*Bacteria_ge*	✓	✓	✓		✓	✓
	✓	✓	✓			✓
	✓			✓		✓
	✓					✓
	✓					
*Bacteroidales_ge*	✓			✓		✓
	✓					✓
	✓					
*Bacteroides*	✓			✓		✓
	✓					✓
*Bradymonadales_ge*	✓					✓
*Campylobacter*	✓					
*Candidatus_Saccharimonas*	✓					✓
*Candidatus_Soleaferrea*	✓					✓
	✓					
*Catenibacillus*	✓					✓
	✓					
*Christensenellaceae_R.7_group*	✓			✓		✓
	✓					✓
	✓					
*Clostridia_ge*	✓			✓		✓
	✓					✓
	✓					
						✓
*Clostridiaceae_ge*	✓					✓
	✓					
*COB_P4.1_termite_group_ge*	✓			✓		✓
	✓					✓
*Endomicrobium*	✓					✓
*Fibrobacter*	✓					✓
	✓					
*Firmicutes_ge*	✓			✓		✓
	✓					✓
	✓					
*Gastranaerophilales_ge*	✓					✓
*Lachnospirales_ge*	✓					✓
	✓					
*Muribaculaceae_ge*	✓			✓		✓
	✓					✓
*NK4A214_group*	✓			✓		✓
	✓					✓
	✓					
*Oligosphaeraceae_ge*	✓					✓
*Oscillospiraceae_ge*	✓			✓		✓
	✓					✓
	✓					
*Oscillospirales_ge*	✓			✓		✓
	✓					✓
	✓					
*Phascolarctobacterium*						
*Prevotella*	✓			✓		✓
	✓					✓
	✓					
*Prevotellaceae_UCG-001*	✓			✓		✓
	✓					✓
	✓					
*Prevotellaceae_UCG-003*	✓			✓		✓
	✓					✓
	✓					
*RF39_ge*	✓					✓
*Selenomonadaceae_ge*	✓					✓
	✓					
*Streptococcus*	✓					✓
*Treponema*	✓					✓
	✓					
*Verrucomicrobiota_ge*	✓	✓	✓			✓
	✓			✓		✓
	✓					✓

Unfilled rows within a bacterial genus represent one or more OTUs of this genus that do not possess any of the orthologues.

## Data Availability

Raw amplicon sequencing libraries were submitted to the NCBI database under bioproject number PRJNA1170059.

## Appendix A

**Table A1:** Serum concentrations of hypoglycin A (µmol/L) and MCPA-carnitine (nmol/L).

		CONTROL	CoG	AM-S	AM-NS	Diseased Horses
HGA	Mean ± SD	<LD	1.46 ± 1.81 ^a^	4.59 ± 3.67	7.32 ± 5.61	6.01 ± 4.88 ^a^
	CI [LLCI-ULCI]		[0.00–3.05]	[2.52–6.67]	[4.28–10.37]	[3.88–7.72]
MCPA-carnitine	Mean ± SD	<LD	9.63 ± 14.03 ^b^	369.62 ± 972.82 ^c^	507.73 ± 485.45 ^c^	441.44 ± 746.02 ^b^
	CI [LLCI-ULCI]		[0.00–21.92]	[0.00–920.04]	[243.84–771.62]	[149–733.88]

HGA: Hypoglycin A/MCPA-carnitine: methylenecyclopropylacetyl-carnitine/< LD = below the limit of detection (0.09 μmol/L for HGA and 0.01 nmol/L for MCPA-carnitine), SD = standard deviation, CI = Confidence interval, LLCI = Lower Limit of Confidence Interval, ULCI = Upper Limit of Confidence Interval. The groups represented are control horses (CONTROL), cograzers (CoG), survivors (AM-S) and non-survivors (AM-NS) of atypical myopathy and diseased horses. a—Significant difference in mean serum concentration of HGA, *p* < 0.01. b—Significant difference in mean serum concentration of MCPA-carnitine, *p* < 0.0001. c—Significant difference in mean serum concentration of MCPA-carnitine, *p* < 0.05.

**Table A2:** Serum concentrations of selected acylcarnitines (μmol/L).

		CONTROL	CoG	AM-S	AM-NS	Diseased Horses
C2	Mean ± SD	7.43 ± 0.96	6.59 ± 2.15	23.87 ± 14.64 *	43.05 ± 25.42 *	33.84 ± 22.74 *
	CI [LLCI-ULCI]	[6.67–8.39]	[4.71–8.73]	[15.58–38.51]	[29.23–68.47]	[24.93–56.58]
C4	Mean ± SD	0.65 ± 0.28	0.85 ± 0.43	10.76 ± 7.02 *	21.06 ± 15.16 *	16.12 ± 12.85 *
	CI [LLCI-ULCI]	[0.43–0.93]	[0.48–1.28]	[6.79–17.78]	[12.81–36.22]	[11.08–28.97]
C5	Mean ± SD	0.29 ± 0.05	0.93 ± 0.51 *	9.52 ± 7.83 *	21.64 ± 14.15 *	15.82 ± 12.90 *
	CI [LLCI-ULCI]	[0.25–0.33]	[0.48–1.44]	[5.10–17.35]	[13.94–35.79]	[10.77–28.72]
C10	Mean ± SD	0.01 ± 0.00	0.04 ± 0.03 *	0.55 ± 0.54 *	0.96 ± 1.39 *	0.77 ± 1.07 *
	CI [LLCI-ULCI]	[0.01–0.01]	[0.01–0.07]	[0.25–1.10]	[0.21–2.35]	[0.35–1.84]
C12:1	Mean ± SD	0.01 ± 0.00	0.01 ± 0.01	0.20 ± 0.13 *	0.28 ± 0.26 *	0.24 ± 0.21 *
	CI [LLCI-ULCI]	[0.00–0.01]	[0.00–0.02]	[0.13–0.33]	[0.14–0.55]	[0.16–0.45]
C14	Mean ± SD	0.01 ± 0.00	0.05 ± 0.07	0.22 ± 0.13 *	0.34 ± 0.28 *	0.28 ± 0.23 *
	CI [LLCI-ULCI]	[0.01–0.02]	[0.00–0.12]	[0.14–0.35]	[0.19–0.62]	[0.19–0.51]
C14:1	Mean ± SD	0.01 ± 0.00	0.03 ± 0.01	0.33 ± 0.22 *	0.54 ± 0.56 *	0.44 ± 0.43 *
	CI [LLCI-ULCI]	[0.01–0.01]	[0.01–0.04]	[0.21–0.55]	[0.24–1.10]	[0.27–0.87]
C18:1	Mean ± SD	0.04 ± 0.02	0.08 ± 0.06	0.61 ± 0.48 *	0.92 ± 0.86 *	0.77 ± 0.71 *
	CI [LLCI-ULCI]	[0.03–0.06]	[0.02–0.14]	[0.34–1.09]	[0.46–1.78]	[0.49–1.48]

* Mean over the percentile 99 of reference range obtained with control horses, SD = standard deviation, CI = Confidence interval, LLCI = Lower Limit of Confidence Interval, ULCI = Upper Limit of Confidence Interval. The groups represented are control horses (CONTROL), cograzers (CoG), survivors (AM-S) and non-survivors (AM-NS) of atypical myopathy and diseased horses.
